# Decoding a novel non-enzymatic protein acetylation mechanism in sperm that is essential for fertilizing potential

**DOI:** 10.1186/s40659-025-00613-6

**Published:** 2025-05-29

**Authors:** María Iniesta-Cuerda, Jan Nevoral, Dario Krapf, Julián Garde, Ana Josefa Soler-Valls, Marc Yeste

**Affiliations:** 1 Biotechnology of Animal and Human Reproduction (TechnoSperm), Institute of Food and Agricultural Technology, University of Girona, 17003 Girona, Spain; 2 Unit of Cell Biology, Department of Biology, Faculty of Sciences, University of Girona, 17003 Girona, Spain; 3 Biomedical Center, Faculty of Medicine in Pilsen, Charles University, Alej Svobody 76, 323 00 Pilsen, Czech Republic; 4 Department of Histology and Embryology, Faculty of Medicine in Pilsen, Charles University, Alej Svobody 76, 323 00 Pilsen, Czech Republic; 5 Laboratory of Cell Signal Transduction Networks, Instituto de Biología Molecular y Celular de Rosario (IBR), CONICET-UNR, Blvd. 27 de Febrero 210 Bis, S2000EZP Rosario, Argentina; 6 Laboratory of Reproductive Medicine, Faculty of Biochemical and Pharmaceutical Sciences, National University of Rosario, Suipacha 531, S2002LRK Rosario, Argentina; 7 SaBio IREC (CSIC-UCLM-JCCM), Campus Universitario, 02071 Albacete, Spain; 8 Catalan Institution for Research and Advanced Studies (ICREA), 08010 Barcelona, Spain

**Keywords:** Non-enzymatic acetylation, pH, Sperm capacitation, Acrosome reaction, Motility, α-tubulin

## Abstract

**Background:**

Protein acetylation has emerged as essential for sperm function, attracting considerable attention recently. Acetylation, typically mediated by lysine acetyltransferases, involves attaching an acetyl group from acetyl-coenzyme A to lysine residues in proteins. Under alkaline conditions, however, acetylation can occur with minimal enzymatic involvement, primarily due to an elevated pH. As sperm migrate towards the ampulla, they experience increasing intracellular pH (pHi) while undergoing two crucial processes for fertilization: capacitation and the acrosome reaction (AR). Whereas the involvement of acetylating enzymes in these events has been partially investigated, the potential for non-enzymatic acetylation driven by the pHi alkalinization remains unknown.

**Results:**

This study examined protein acetylation (acLys) levels in sperm incubated under capacitating conditions at pH 7.2 and pH 9.0, the latter condition potentially promoting non-enzymatic acetylation. To more precisely investigate the occurrence of non-enzymatic acetylation events, acetyltransferase activity was selectively attenuated using a specific cocktail of inhibitors. The functional implications of these conditions were assessed by examining key fertilization-related sperm attributes, including motility during capacitation and the ability to initiate the AR. Results demonstrated that alkaline conditions elevated basal acLys levels even with reduced acetyltransferase activity (*P* < 0.05), indicative of non-enzymatic acetylation. α-tubulin, particularly in the midpiece of the sperm flagellum, was identified as a specific target of this modification, correlating with diminished motility during capacitation. Following the AR, acLys levels in the head and midpiece decreased (*P* < 0.05) under conditions promoting non-enzymatic acetylation, accompanied by reductions in intracellular and acrosomal pH. In contrast, acLys levels and pH in the sperm head incubated under standard capacitating conditions (pH 7.2) remained stable. Sperm exposed to conditions conducive to non-enzymatic acetylation exhibited an impaired ability to trigger the AR (*P* < 0.05) compared to those maintained at pH 7.2. Notably, diminished acetylase activity emerged as a key factor impairing the maintenance of intracellular and acrosomal pH levels attained during capacitation, even under a pH of 9.0.

**Conclusion:**

This study provides novel evidence for the occurrence of non-enzymatic acetylation in sperm, linked to the modulation of α-tubulin acetylation levels and motility during capacitation. Additionally, it suggests that acetyltransferase activity may play a crucial role in regulating intracellular and acrosomal pH levels in capacitated sperm, facilitating the AR.

**Supplementary Information:**

The online version contains supplementary material available at 10.1186/s40659-025-00613-6.

## Introduction

Acetylation is one of the most prevalent and crucial posttranslational modifications (PTMs) of proteins in cells, often rivaling—or even surpassing—phosphorylation in frequency. While this is particularly prominent in bacteria, it is not confined to them, as similar levels of abundance have been observed in eukaryotic organisms, especially within the mitochondrial environment [1–3]. Hence, acetylation is integral to a wide array of signaling pathways, and its reversible nature offers cells a dynamic for effectively regulating these cascades. During acetylation, the acetyl group from acetyl coenzyme A (Ac-CoA) is transferred to the primary amine located in the ε-position of a lysine side chain, in a given polypeptide sequence. While this transfer is primarily catalyzed by acetyltransferases (KATs), some acetylations occur non-enzymatically via direct interaction of proteins with Ac-CoA, with both mechanisms being balanced by the activity of deacetylases (KDACs).

Whereas acetylation mediated by KATs has been extensively explored, with comprehensive acetylome studies revealing a vast array of acetylated proteins in varied cellular compartments (revised in [2, 4]), research on non-enzymatic acetylation has lagged behind. The concept of non-enzymatic acetylation began with observations of peptide acetylation in the presence of Ac-CoA [5]. Over time, it has gained attention from studies in yeast, mice, and growth-arrested cells, which showed that acetylation changes with the varying Ac-CoA levels resulting from metabolic interventions [6, 7]. The proposed mechanism for non-enzymatic acetylation involves the direct transfer of acetyl groups to protein amino groups [8]. This process occurs naturally at alkaline pH, where lysine residues are deprotonated [9], allowing acetyl-CoA to transfer its acetyl group without the need for enzymatic action [10]. Consequently, alkaline pH conditions increase the proportion of deprotonated lysines which, together with high concentrations of acetyl-CoA, enhance non-enzymatic acetylation [5].

In sperm, the pH of the extracellular milieu (pHe) decides for cell physiology [11]. The original developmental conditions for sperm are quite different from those the male gametes encounter while traveling to the oocyte. In effect, while stored in the epididymis, the acidic pHe decelerates metabolism, preventing premature activation and conserving energy [12]. Upon ejaculation, sperm experience rising pHe levels throughout their journey to the oviduct where they complete capacitation [13–16], the final maturation step required for fertilization [17]. During capacitation, sperm undergo a significant alkalinization of their intracellular pH (pHi) along with an important increase in their metabolism [18, 19], conditions that might favor the occurrence of non-enzymatic acetylation events. If this were the case, non-enzymatic acetylation events would be expected to contribute to the sperm acetylome landscape at fertilization. Noteworthy, lysine acetylation has previously been suggested to be essential in the development of hyperactivated motility and acrosomal responsiveness in mouse sperm [20].

In this context, the aims of this study were: (1) to determine whether non-enzymatic acetylation occurs in sperm, and (2) to evaluate its potential contribution to the sperm acetylome under capacitating conditions. We further investigated whether changes in acetylation dynamics are functionally linked to intracellular pHi regulation preceding the acrosome reaction (AR). Using the pig as a model, we pharmacologically inhibited sperm acetyltransferases to slow down enzymatic acetylation, and employed alkaline pHe to induce the transfer of Ac-CoA to proteins. Under these conditions, we observed clear evidence of non-enzymatic acetylation, with α-tubulin being identified as a potential motility-related target during capacitation, and indications that acetyltransferases may participate in pHi regulation prior to the AR.

## Materials and methods

### Reagents

All chemicals were sourced from Sigma Aldrich (Barcelona, Spain) unless otherwise indicated.

### Samples and experimental design

Ethical considerations were rigorously observed throughout the study, with all animal procedures being conducted in strict compliance with current European Union legislation on animal health, welfare, and husbandry (Directive 2010/63/EU). Semen samples were sourced from Grup GePork (Les Masies de Roda, Spain), a commercial artificial insemination (AI) center that adheres to European and Spanish regulations governing the commercialization of AI doses and animal welfare. Semen donors included four healthy, fertile, and mature boars (1.5–3 years), and samples were collected using the gloved-hand method. The semen was diluted to a concentration of 33 × 10^6^ sperm/mL in Duragen extender (Magapor, Spain) and maintained at 17 °C before being delivered to the laboratory within 4 h after collection.

Upon arrival, the samples from the four boars were pooled and centrifuged to remove the extender and seminal plasma (600×*g*, 16 °C for 5 min). The resulting pellets were resuspended in either a non-capacitating medium (NCAP: 96 mM NaCl, 4.7 mM KCl, 0.4 mM MgSO_4_ × 7 H_2_O, 0.3 mM Na_2_HPO_4_ × H_2_O, 5.5 mM glucose, 21.6 mM sodium l-lactate, 1 mM sodium pyruvate, 10 mM Na_2_HPO_4_, and 20 mM HEPES) or a capacitating medium (CAP; consisting of NCAP medium supplemented with 0.5 mM CaCl_2_ × 2 H_2_O, 10 mM NaHCO_3_, and 3 mg/mL bovine serum albumin, BSA). Additionally, a third group was prepared to investigate non-enzymatic acetylation by adding a cocktail of acetyltransferase inhibitors (Cocktail) to the capacitating medium at varying concentrations previously shown to effectively modify specific acetyltransferases identified in porcine sperm [21]. These inhibitors were: WM 1119 (MYST family acetyltransferase inhibitor, 1 µM, Sigma-Aldrich SML3067; [22]), Anacardic acid (Histone acetyltransferase inhibitor, 1 µM, Sigma-Aldrich A7236; [23]); NU9056 (KAT 5 inhibitor 0.5 µM, Sigma-Aldrich 5.00511; [24]); and A-485 (p300/CBP inhibitor 1 µM, MedChemExpress HY-107455 (Monmouth Junction, New Jersey, USA; [25])). All inhibitors were prepared in DMSO at a concentration not exceeding 0.1% (v/v). To control for vehicle effects, all experimental conditions, including NCAP and CAP samples, were supplemented with 0.1% (v/v) DMSO. Upon media preparation, including cocktail addition and before starting incubations, all media under evaluation were adjusted and buffered to pH levels of 7.2 and 9.0.

After preparation, samples were incubated at 38.5 °C for 3 h to induce capacitation. The AR was triggered by adding 10 µg/mL progesterone to all samples, followed by an additional 10 min of incubation. Samples were evaluated at 3 h and after inducing the AR (3 h + 10 min) for motility, intracellular and acrosomal pH, and phosphorylation of protein kinase A (pPKA) and tyrosine (pY) substrates. Protein acetylation at lysine 40 (acLys) and α-tubulin acetylation (acTub) levels were also assessed. This experimental design was replicated four times.

### Sperm motility analysis

Sperm suspensions were placed into pre-warmed (38 °C) 20-µm Leja chamber slides (Leja Products BV; Nieuw-Vennep, The Netherlands), and motility was examined using a computer-assisted semen analysis system. This setup included a negative phase-contrast microscope (Olympus BX41 with a 10 × 0.30 PLAN objective; Olympus, Tokyo, Japan) and a computer running Integrated Sperm Analysis System software (V1.0; Proiser SL, Valencia, Spain). The analysis parameters were as follows: 25 frames captured and a cell size range of 10–80 μm^2^. In each experiment, a minimum of 500 sperm were examined per replicate, with three technical replicates assessed per sample. The parameters measured included mean path velocity (VAP, μm/sec), curvilinear velocity (VCL, μm/sec), straight-line velocity (VSL, μm/sec), linearity (LIN, %), calculated as LIN = VSL/VCL × 100; straightness (STR, %), determined by VSL/VAP × 100; and the motility parameter wobble (WOB, %), derived from VAP/VCL × 100. Sperm were considered motile when their VAP was equal to or greater than 10 μm/sec, and progressively motile when their STR was equal to or greater than 45%.

### pH measurements

For the evaluation of intracellular and acrosomal pH, the pH-sensitive dye BCECF-AM (Invitrogen, Waltham, Massachusetts, USA) and Lysosensor™ green DND-189 (Thermo Fisher Scientific, Massachusetts, USA) were respectively employed. To distinguish the viable sperm population, samples were incubated with the Live/Dead™ Fixable violet dye (L/D; Thermo Fisher Scientific) according to the manufacturer’s instructions. Briefly, 1 μL of reconstituted L/D dye was added to 1 × 10^6^ of sperm at 38 °C for 30 min. The viable sperm population corresponded to those cells with dimmer L/D signal. Subsequently, sperm were incubated with 2.5 µM of BCECF-AM and with 1 µM of Lysosensor™ green DND-189 at 38 °C for 10 min, and then washed once at 600×*g* for 5 min and resuspended in the appropriate NCAP or CAP media. L/D, BCECF-AM and Lysosensor™ fluorescences were measured using a CytoFLEX cytometer (Beckman Coulter; Fullerton, California, USA), with excitation at 405 and 488 nm and emission wavelengths of: (i) L/D at 451 nm (detected by the PB450 channel (450/45)); (ii) BCECF-AM shifting from 525 nm (detected by the FITC channel (525/40)) to 640 nm (detected through the PC5.5 channel (690/50)); (iii) Lysosensor™ at 525 nm (detected by the FITC channel (525/40)). Once BCECF-AM enters the cell, nonspecific esterases convert it from its nonfluorescent form to a fluorescent indicator sensitive to pH changes. The 525/640 ratio obtained by the BCECF emission increased with pHi, as Chow and Hedley [26] outlined. The fluorescence ratio relative to pHi was calibrated for each replication using the K^+^/H^+^ ionophore nigericin (Sigma-Aldrich, N7143) to equilibrate the pH gradient across the plasma membrane. A high-concentration K^+^ buffer (120 mM KCl, 2 mM CaCl_2_, 20 mM Trizma Base, 11 mM d-glucose, 1 mM sodium pyruvate, and 50 mM HEPES) was utilized across a range of external pH values (6.5 to 10). The fluorescence ratios were plotted against these known pH values, and the pHi of sperm samples was determined by the interpolation of the resulting standard curve. As Lysosensor™ labels acidic organelles, such as the acrosome of viable sperm, the decrease of fluorescence upon alkalinization was recorded. Both intracellular and acrosomal pH values are presented as normalized to those of NC, which were assigned a reference value of 1.

### Immunofluorescence

Sperm proteins were analyzed using immunofluorescence (IF) through slightly adapted protocols from previous studies [27]. Initially, sperm were stained with L/D to identify viable sperm populations. Subsequently, sperm were fixed, permeabilized, and blocked using PBS containing paraformaldehyde (PFA,4%, w/v), Triton-X 100 (0.1%, v/v), Tween-20 (0.2%, v/v), and BSA (5%, w/v), followed by incubation with primary antibodies (1:200, v/v): mouse anti-pY (Sigma-Aldrich 05-321X), rabbit anti-pPKA (Cell Signaling mAb #9624 (Danvers, Massachusetts, USA)); mouse anti-acLys (Abcam ab22550 (Cambridge, UK)), and rabbit anti-acTub (Cell Signaling mAb #5335). Two primary antibody cocktails were used: one containing anti-pY and anti-pPKA antibodies, and the other containing anti-acLys and anti-acTub antibodies. Detection was carried out using a cocktail of secondary antibodies (anti-mouse and anti-rabbit Alexa Fluor 488 and 647, Abcam), and non-specific binding was assessed by omitting primary antibodies in additional control samples. Evaluation and imaging were performed with a FlowSight flow cytometer AMNIS® (Merck, Darmstadt, Germany) equipped with three lasers at excitation wavelengths of 405 (10 mW), 488 (30 mW) and 785 nm (10 mW), and a 10 × microscope objective. The brightfield and emission wavelengths of specific markers were acquired with the following channels: two brightfield channels (channels 1 and 9.0); (i) side scatter emission (SSC; channel 6); (ii) L/D at 451 nm (detected by the channel 7); (ii) Alexa Fluor 488 at 521 nm (detected by the channel 2); (iii) Alexa Fluor at 647 nm and 673 nm (detected by the channel 11). Signal intensities and percentages of the markers under study in viable sperm were recorded with the IDEAS® software (AMNIS EMD Millipore). The sperm population was gated using aspect ratio and area parameters, and a minimum of 10,000 sperm events were examined. Then, standard focus and single-cell gating were applied as per the IDEAS® software guidelines (Supplementary material, Fig. 1). Additionally, a mask analyzing system for evaluating specific marks in sperm regions was employed as previously described [28] with slight modifications (for more details, see Supplementary material, Fig. 1). This mask-based evaluation approach provided precise data for specific phosphorylation and acetylation marks by plotting pY, pPKA, acLys and acTub positive sperm (%), and intensities (mean fluorescence intensity, MFI) within the masked region (Supplementary material, Fig. 1). The accumulations of pY, pPKA, acLys, and acTub proteins—as measured by the mean fluorescence intensity (MFI) of the corresponding antibodies—were normalized to the negative control (NC), which was assigned a reference value of 1.

### Immunoblotting

Immunoblotting was carried out according to an established protocol with minor adjustments [27]. Sperm pellets (approximately 5000 cells) were resuspended in RIPA lysis buffer (50 mM Tris HCl, 150 mM NaCl, 0.5% (w/v) sodium deoxycholate, 1.0 mM EDTA, 0.1% (w/v) SDS and 0.01% (w/v) sodium azide at a pH of 7.4), which was supplemented at a ratio of 1:100 (v/v) with a protease inhibitor cocktail containing sodium orthovanadate (700 mM) and phenylmethanesulfonyl fluoride (0.1 mM). Samples were subjected to ultrasonic treatment in a sonicator (Selecta group, Barcelona, Spain) on ice for 5 min (five pulses each; 20 kHz), followed by incubation at 4 °C for 30 min. Subsequently, samples were centrifuged at 10,000×*g* and 4 °C for 15 min to collect the supernatant. Protein extracts were diluted in Laemmli reducing buffer (5 ×) containing 5% (v/v) β-mercaptoethanol, heated at 95 °C for 5 min, and loaded onto 8–12% polyacrylamide gels. Electrophoresis was run at 20 mA and 120–150 V, after which proteins were transferred onto polyvinylidene fluoride membranes. Membranes were blocked with T-TBS containing 5% (w/v) BSA before being incubated overnight with primary antibodies diluted in T-TBS as follows: anti-pY (1:10,000; Sigma-Aldrich 05-321X), anti-phospho-pPKA-substrates (1:2000; Cell Signaling mAb #9624), anti-acLys (1:500; Abcam ab22550), or anti-acTub (1:1000; Cell Signaling mAb #5335). Secondary antibodies were diluted 1:10,000 (v/v) in T-TBS and incubated at room temperature for 1 h. Protein detection was performed using a chemiluminescent substrate (WesternSure® PREMIUM, LI-COR (Lincoln, Nebraska, USA)), and membranes were scanned on a C-DiGit Chemiluminescent Western Blot Scanner (LI-COR). Images of membranes were processed using Image Studio Lite V5 software (LI-COR), and the integrated density of the bands signal was quantified. Membranes were stripped through incubation in a stripping buffer containing 2% SDS, 0.74% β-mercaptoethanol and 62.5 mM Tris (pH 6.5) at 60 °C for 15 min, followed by six washes of 5 min each in T-TBS. α-tubulin was used as a loading control, and band intensities were normalized to α-tubulin levels. After normalization, protein band values were standardized to those of the NC, which was assigned a reference value of 1. Molecular masses of proteins were expressed in kilodaltons (kDa).

### Acrosomal integrity evaluation

In order to evaluate the occurrence of the AR, a protocol described elsewhere was followed [27]. In brief, after incubation under the specified conditions for 3 h, 10 µg/mL progesterone was added, followed by a further incubation of 10 min. Sperm were initially stained with L/D to identify the viable populations; samples were then subjected to fixation and permeabilization in PBS containing 4% PFA, 0.1% Triton X-100, and 0.2% Tween-20, with agitation at room temperature for 30 min. Sperm were thereafter incubated with PBS containing 1% BSA and 2.5 µM FITC-labeled peanut agglutinin (PNA-FITC Thermo Fisher Scientific) lectin at room temperature for 1 h. Following this incubation, samples were centrifuged at 850×*g* for 5 min to remove any excess stain. The acrosomal status of sperm was subsequently analyzed using a CytoFLEX flow cytometer with excitation wavelengths of 405 and 488 nm, whereas emissions were measured at 451 nm for L/D (detected through the PB450 channel, 450/45), and 525 nm for PNA-FITC (detected via the FITC channel, 525/40). This analysis identified four distinct sperm subpopulations: (1) viable sperm with an intact acrosome (PNA-FITC^+^/L/D^−^); (2) viable sperm with an exocytosed acrosome (PNA-FITC^−^/L/D^−^); (3) non-viable sperm with an intact acrosome (PNA-FITC^+^/L/D^+^); and (4) non-viable sperm with an exocytosed acrosome (PNA-FITC^−^/L/D^+^). For data presentation, only viable sperm with an intact acrosome (PNA-FITC^+^/L/D^−^) were considered.

### Statistical analyses

Data from four replicates were analyzed using IBM SPSS for Windows Ver. 27.0 (IBM Corp.; Armonk, New York, USA). Normal distribution of data was tested using the Shapiro–Wilk test, and homogeneity of variances was evaluated with the Levene test. For data that fitted with parametric assumptions, a repeated measures ANOVA (intra-subjects factor: incubation time; inter-subjects factor: treatment) followed by a post-hoc Tukey test for pair-wise comparisons was run. Additionally, a one-sample t-test was employed to compare intracellular pH values at the end of the incubation period with the initial pH of each sample (approximately 7.2). When data did not comply with parametric assumptions, Kruskal–Wallis and Wilcoxon tests were run as non-parametric alternatives. Correlations were calculated with the Spearman test.

For clustering sperm based on kinematic features, principal component (PC) analysis (PCA) was performed as described by [29]. Kinematic parameters were reduced to two PC that accounted for 82.29% of the total variance. The first PC was associated to VCL, VSL, VAP, ALH and BCF (a^2^_ij_ values: 0.98, 0.81, 0.89, 0.85 and 0.58, respectively), whereas the second one was related to LIN, STR and WOB (a^2^_ij_ values: 0.97, 0.82 and 0.87, respectively). These regression scores were used as input variables for hierarchical cluster analysis, employing the squared Euclidean distance and the Ward method to classify sperm into two subpopulations: Subpopulation I and Subpopulation II (abbreviated as SPI and SPII). The percentage of sperm in each subpopulation was evaluated through repeated measures ANOVA under the conditions specified above.

Statistical significance was defined as a *P*-value equal to or less than 0.05.

## Results

### The accumulation of acetylated proteins during capacitation relies on both acetyltransferases’ activity and extracellular pH

**Fig. 1:**
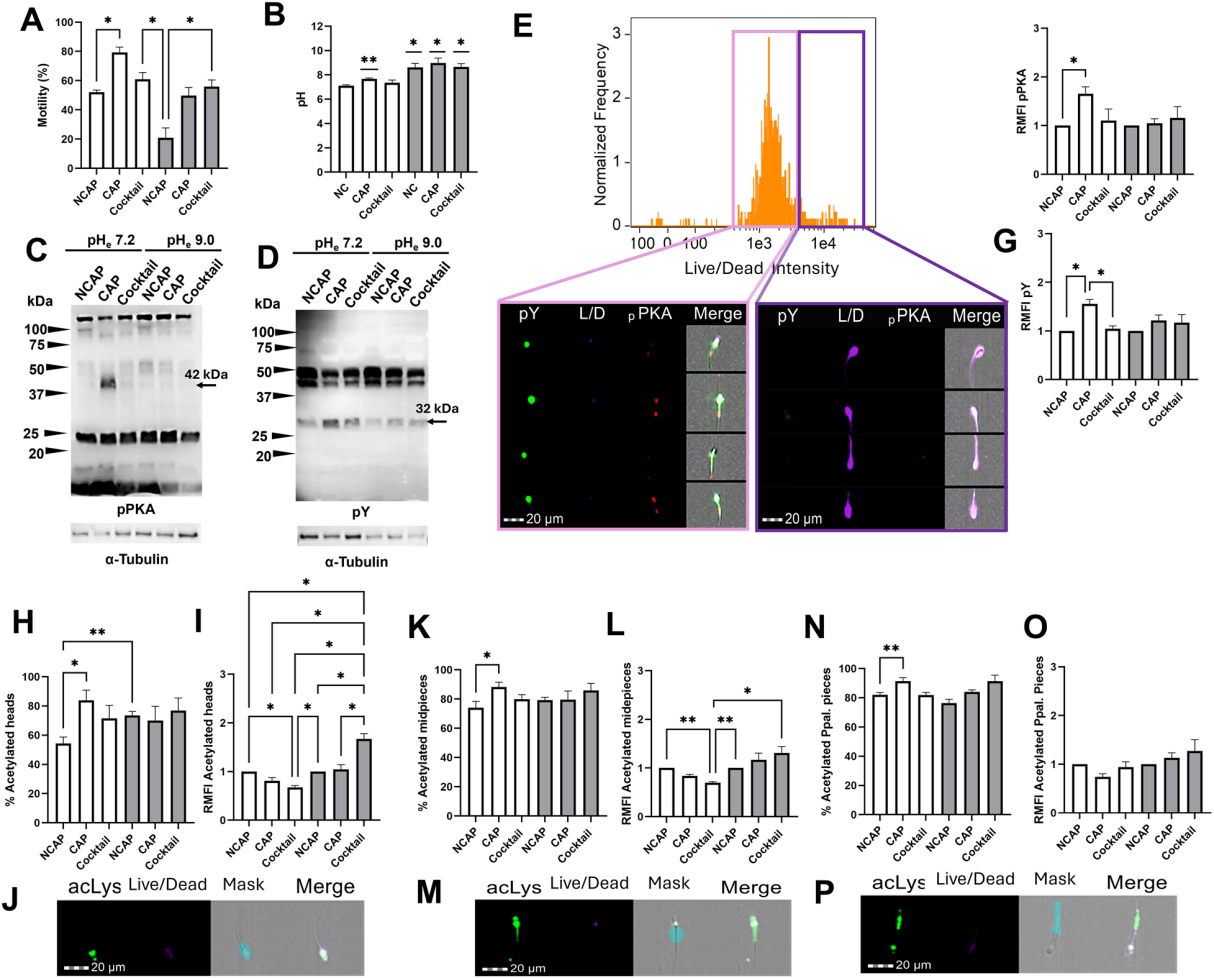
Capacitation achievement, interplay between acetylases activity and extracellular pH (pHe), and evidence of non-enzymatic acetylation events in sperm. Sperm were incubated under non-capacitating (NCAP) and capacitating (CAP) conditions, as well as under capacitating conditions with the acetylase inhibitor cocktail (Cocktail) at pH 7.2 (white bars) and 9.0 (grey bars), and **A** sperm motility, **B** intracellular pH (pHi), and phosphorylation of **C** protein kinase A (pPKA) and **D** tyrosine residues (pY) were evaluated. Immunoblotting with anti-pPKA substrates and anti-pY antibodies revealed prominent 42 and 32 kDa protein bands, respectively, in sperm extracts incubated under standard capacitating conditions at pH 7.2 (arrowheads). Complementary IF results are shown, including **E** the histogram of Live/Dead™ Fixable violet dye (L/D) for the differentiation of the viable sperm population and the quantification of **F** pPKA and **G** pY in that specific population. Acetylation levels of sperm expressed as percentages of sperm with acetylated proteins in the **H** head, **K** midpiece, and **N** principal piece, and as accumulation of acetylated proteins (acLys) in these regions (**I**, **L**, **O,** respectively). Representative images of acetylated proteins in the **J** head, **M** midpiece, and **P** principal piece from the flow cytometry AMNIS image unit are provided. The accumulation of **F** pPKA, **G** pY and **I, L, O** acetylated proteins is expressed as the fold change in mean fluorescence intensity (MFI) relative to the NCAP at pH 7.2 and 9.0 (assigned a value of 1; RMFI). All columns show mean ± s.e.m. (* *P* < 0.05, ** *P* < 0.01). The Wilcoxon signed-rank test and one-sample t-test were employed to compare the mean of pHi after incubation with the baseline pHi at the onset of the experiment (i.e. ± 7.2; *P* = 0.05). Scale bar = 20 µm

**Fig. 2:**
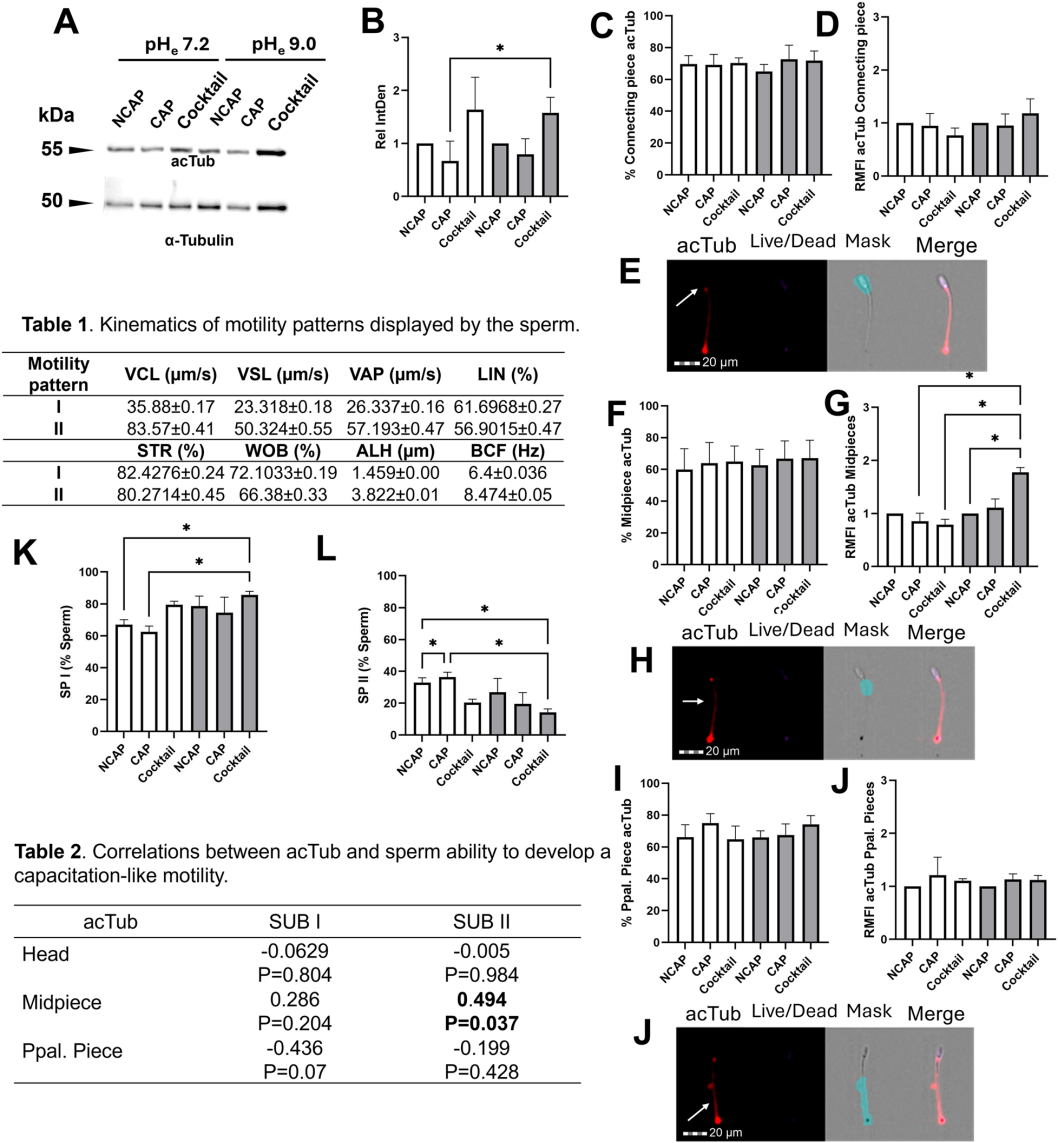
α-Tubulin is targeted by non-enzymatic acetylation, and its accumulation in the midpiece of the flagellum is associated with decreased sperm motility. **A** Immunoblotting with anti-acetylated α-tubulin (acTub) and **B** integrated density (IntDen) of bands signals following incubation under non-capacitating (NCAP) and capacitating (CAP) conditions, as well as under capacitating conditions with the acetylase inhibitor cocktail (Cocktail) at pH 7.2 (white bars) and 9.0 (grey bars). For additional localization details of acTub, immunofluorescence was performed and analyzed using flow cytometry, and the results of the sperm percentage undergoing acTub and its accumulation are shown for the **C, D** head, **F, G** midpiece and **I, J** principal piece of the tail. Representative images of acetylated proteins in the **E** head, **H** midpiece, and **J** principal piece from the flow cytometry AMNIS image unit are provided. The accumulation of acTub is expressed as the fold change in mean fluorescence intensity (MFI) relative to the NCAP sample at each pH (assigned a value of 1; RMFI). Percentage of sperm comprising subpopulations **K** I and **L** II (SPI and SPII) after application of principal component analysis and clustering of sperm based on their kinetics characteristics after capacitation. All columns show mean ± s.e.m. (* *P* < 0.05). Scale bar = 20 µm

While the relevance of the acetylome for sperm capacitation—and particularly the main role of KDACs—was previously demonstrated [20], the potential involvement of KATs has not been described. We therefore aimed to investigate acetylase activity during capacitation, and the potential occurrence of non-enzymatic acetylation under both standard capacitation pH (pH 7.2) and alkaline conditions (pH 9.0). To characterize these mechanisms, we first confirmed that our experimental system induced modifications associated to capacitation. Incubating sperm in a capacitating medium at pH 7.2 significantly enhanced motility compared to non-capacitated controls (*P* < 0.05; Fig. 1A) and induced a rise of pHi compared to the values at the beginning of the experiment (mean ± SEM: 7.86 ± 0.07 vs. 7.18 ± 0.06; *P* < 0.05; Fig. 1B). The initial pHi values for all samples are presented in Table 1 of Supplementary material. Moreover, we identified a prominent 42 kDa band corresponding to a pPKA substrate and another of 32 kDa corresponding to pY protein in extracts of sperm incubated under capacitating conditions; these two bands were absent or weaker, respectively, in non-capacitated samples (Fig. 1C, D). While, based on densitometric analysis, neither the pPKA substrates nor pY levels differed (*P* > 0.05) between capacitated and non-capacitated sperm at pH 7.2 (Fig. 2 Supplementary material), IF analysis on viable sperm using the same antibodies revealed significant differences (*P* < 0.05; Fig. 1E). Accordingly, and in the case of the viable sperm population, pPKAs and pY levels were significantly higher in sperm incubated under capacitating conditions at pH 7.2 than in those incubated in non-capacitating conditions (*P* < 0.05; Fig. 1F, G). Remarkably, such modifications associated with capacitation were not observed when sperm were incubated in a capacitating medium supplemented with a cocktail of acetyltransferase inhibitors (pH 7.2). Under these conditions, motility, pHi, pPKA and pY levels were comparable to those of non-capacitated sperm (Fig. 1A–D).

Incubating sperm in a capacitating medium at pH 9.0 did not alter motility, pPKA or pY levels, which remained comparable to those observed under non-capacitating conditions (Fig. 1A–D). Yet, the incubation of sperm under alkaline conditions induced a rise (*P* < 0.05) of the pHi above that at the beginning of the incubation in all samples (NC: 8.06 ± 0.32, CAP: 8.96 ± 0.41 and Cocktail: 8.66 ± 0.26 *vs* 7.26 ± 0.15). Notably, when sperm were exposed to capacitating conditions at pH 9.0 and in the presence of acetyltransferase inhibitors, motility increased (*P* < 0.05) compared to those incubated in a non-capacitating medium (Fig. 1A).

After evaluating capacitation achievement under the specified conditions, we investigated both the proportion of sperm undergoing acetylation and the accumulation of acetylated proteins in the head, midpiece, and principal piece of viable sperm using IF in combination with flow cytometry. IF results showed a wide distribution of acetylated proteins, mostly in the mid and principal pieces, but also in the head (Fig. 1H–P). Overall, the number of sperm undergoing acetylation of proteins either in the head, midpiece, or principal piece was higher (*P* < 0.05) in capacitated sperm compared to non-capacitated samples, but only under pHe 7.2 (Fig. 1H, K, N). Notably, this increase was mitigated in the presence of the acetyltransferase inhibitors cocktail (Fig. 1H, K, N), confirming the specificity of the inhibitors used in these experiments. Incubating sperm at pH 9.0 increased (*P* < 0.05) acetylation levels compared to those incubated at pH 7.2, independent of capacitation-associated events, as indicated by the proportion of sperm undergoing acetylation under non-capacitating conditions (Fig. 1H). Additionally, sperm incubated under capacitating conditions in the presence of acetylase inhibitors at pH 7.2 showed significantly (*P* < 0.05) lower accumulation of acetylated lysines in the head and midpiece of the tail compared to the NCAP sample at both pH 7.2 and 9.0 (Fig. 1I, L), likely due to the activity of the corresponding deacetylating enzymes [8], further validating the efficacy of our cocktail of inhibitors. Interestingly, hyperacetylation of lysines was observed in the sperm head and midpiece when acetyltransferases were inhibited at pH 9.0; this was not detected in the other samples and those incubated in the same medium but at pH 7.2 (Fig. 1I, L). On the contrary, incubating sperm at pH 9.0 alone did not produce this increase in acLys (Fig. 1I, L), indicating that (i) acetylase inhibitors were effective at pH 9.0, (ii) non-inhibited enzymes were not stimulated by alkaline pH, and (iii) non-enzymatic acetylation occurred when sperm were incubated in an alkaline medium containing acetyltransferase inhibitors. The accumulation of acetylated proteins was observed in all the samples, regardless of the treatment (Fig. 1O).

### Non-enzymatic acetylation of α-tubulin in the midpiece is associated with a failed transition to hyperactivated motility during capacitation

Α-tubulin is essential for sperm motility [30] mainly because it is the principal component of the flagellum axoneme (Eddy, 2006), while being a primary substrate for acetylation. Whether the acetylation observed during capacitation specifically targets α-tubulin as a mechanism for regulating sperm motility, however, has not been previously investigated. To address this, we first evaluated global acTub levels within the connecting piece, midpiece, and principal piece of the flagellum. Then, we worked out correlations between acTub levels and specific patterns of sperm motility, based on differential kinematic features. Western blotting employing the anti-acetylated Lys40 α-tubulin (acTub) antibody demonstrated that α-tubulin is not one of the proteins undergoing acetylation during capacitation, as similar acTub levels were found between sperm incubated in a capacitating medium and those incubated in a non-capacitating medium, regardless the pHe (Fig. 2A, B). Despite this, incubating sperm under capacitating conditions at pH 9.0 in the presence of acetyltransferase inhibitors (which promoted non-enzymatic acetylation, as shown in Fig. 1C) resulted in a significant accumulation of acTub (*P* < 0.05) in comparison to that observed under regular capacitating conditions (CAP 7.2). Complementary IF assays revealed that acTub mainly accumulated in the midpiece region of viable sperm (Fig. 2G). Additionally, IF showed that viable sperm incubated in a capacitating medium containing acetylase inhibitors accumulated more acTub at pH 9.0 than at pH 7.2 and under non-capacitating conditions at pH 9.0 (*P* < 0.05; Fig. 2G). Further significant differences in the percentage of sperm showing acTub, or in the acTub accumulation within the distinct regions of viable sperm were not found between treatments, in line with Western blot results (Fig. 2D–J).

We further investigated the relevance of acTub by correlating the acTub levels with sperm motility. First, we ran a PCA involving all the kinematic parameters, followed by hierarchical clustering to identify the specific motility patterns exhibited by the different sperm subpopulations within each sample. Two distinct motility patterns were identified (Pattern I and II), leading to the classification of sperm into two differentiated subpopulations (SPI and SPII), with the associated motility descriptors presented in Table 1. Briefly, the sperm population displaying pattern I (SPI) exhibited the slowest motility, showing the smallest values for VCL, VAP and VSL, coupled with a reduced lateral motion (lowest amplitude of ALH), and enhanced linear progression (relatively high STR, WOB, and LIN values). In contrast, the sperm population displaying pattern II (SPII) included those sperm with the highest values for speed (VCL, VAP, and VSL) and vigorous flagellar action (as indicated by the highest BCF and ALH), but with reduced forward progression (lower LIN, STR, and WOB compared to SPI) and more vigorous flagellar bending (indicated by the highest BCF), which could correspond with the hyperactivated motility pattern previously described [31]. Subsequently, we evaluated the distribution of sperm into these two subpopulations after incubation under the conditions of the study. The percentage of sperm in SPI remained consistent between non-capacitated and capacitated samples, irrespective of the pH (Fig. 2K). In contrast, SPII significantly increased (*P* < 0.05) when sperm were incubated under capacitating conditions at pH 7.2 compared to non-capacitated samples, supporting the notion that a sperm population exhibiting a hyperactivated-like motility pattern emerges after incubation under capacitating conditions. Acetylase inhibition (cocktail) at pH 7.2 suppressed that variation in SPII. Notably, under non-enzymatic acetylation conditions, the sperm subpopulation exhibiting hyperactivated-like motility (SPII) was significantly lower (*P* < 0.05) than in capacitating conditions at pH 7.2. Similarly, the subpopulation with slower, progressive movement (SPI) was significantly reduced (*P* < 0.05; Fig. 2L). This suggests that the increase in acTub observed under conditions leading to non-enzymatic acetylation compromises the transition to hyperactivated-like motility. Notably, similar trends were observed when comparing the results to the non-capacitated sample (*P* < 0.05; Fig. 2K–L), indicating the strong influence of this type of acetylation as, not only did it interrupt the transition to hyperactivation, but it also abolished the motility characteristics observed in non-capacitated samples. To better address the function of non-enzymatic acetylation, we calculated the correlation between acTub levels and the percentages of SPI and SPII during capacitation. As depicted in Table 2, the percentages of sperm accumulating acTub in the midpiece were correlated with the percentages of those belonging to SPII (r = 0.494). This suggests that the increase in acTub observed under capacitating conditions leading to non-enzymatic acetylation may underlie the disrupted transition to hyperactivated-like motility and help maintain sperm in a state even more deactivated than the non-capacitated one.

### The AR occurs with the accumulation of acLys proteins in the head, and with alkaline intracellular and acrosomal pH

**Fig. 3:**
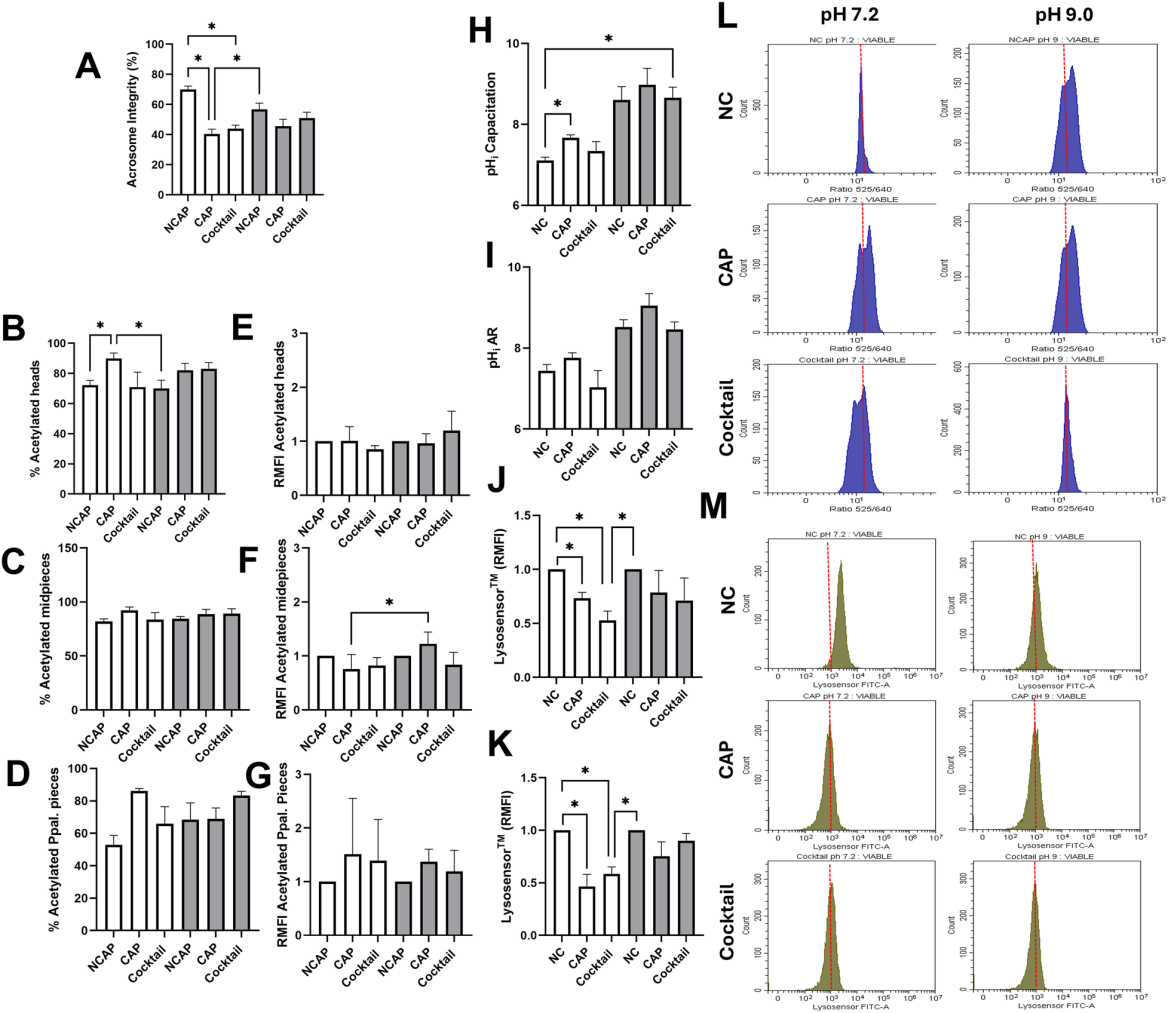
Evaluation of protein acetylation after inducing the AR. Following incubation in non-capacitating (NCAP) and capacitating (CAP) conditions, as well as under capacitating conditions with the acetylase inhibitor cocktail (Cocktail) at pH 7.2 (white bars) and 9.0 (grey bars), sperm were exposed to progesterone (10 µM/mL–10 min) to induce the AR. First, acrosome integrity was evaluated to corroborate the occurrence of the AR in the conditions under study. **A** Percentages of viable sperm exhibiting an intact acrosome assessed by FITC-PNA staining and flow cytometry. Acetylation levels in sperm expressed as percentage of viable sperm with acetylated proteins in the **B** head, **C** midpiece, and **D** principal piece, and as accumulation of acetylated proteins in these regions (**E**, **F**, **G**, respectively). The accumulation of acetylated proteins is expressed as the fold change in mean fluorescence intensity (MFI) relative to the NCAP sample at each pH (assigned a value of 1; RMFI). Intracellular and acrosomal pH of sperm incubated in the conditions under study after capacitation (**H, K**, respectively), and the AR (**I, J**, respectively). As alkalinization of acrosomal pH led to a decrease in Lysosensor™, the acrosomal pH is expressed as the fold change in mean fluorescence intensity (MFI) of the Lysosensor™ fluorochrome relative to the NCAP sample at each pH (assigned a value of 1; RMFI). Representative histograms of **L** BCECF-AM and **M** Lysosensor™ intensities assessed by flow cytometry. BCECF-AM fluorescence shifted from 525 to 640 nm in a pH-dependent manner; thus, BCECF-AM intensity is expressed as 525/640 nm ratio. Incubation in CAP at pH 7.2 resulted in the appearance of a sperm subpopulation with a more alkaline pH compared to those incubated in NC at pH 7.2. This subpopulation exhibited a higher 525/640 nm ratio, appearing to the right of the main population. As Lysosensor™ labels acidic organelles such as the acrosome, a decrease in its intensity reflects alkalinization. All columns show mean ± s.e.m. (* *P* < 0.05)

**Fig. 4:**
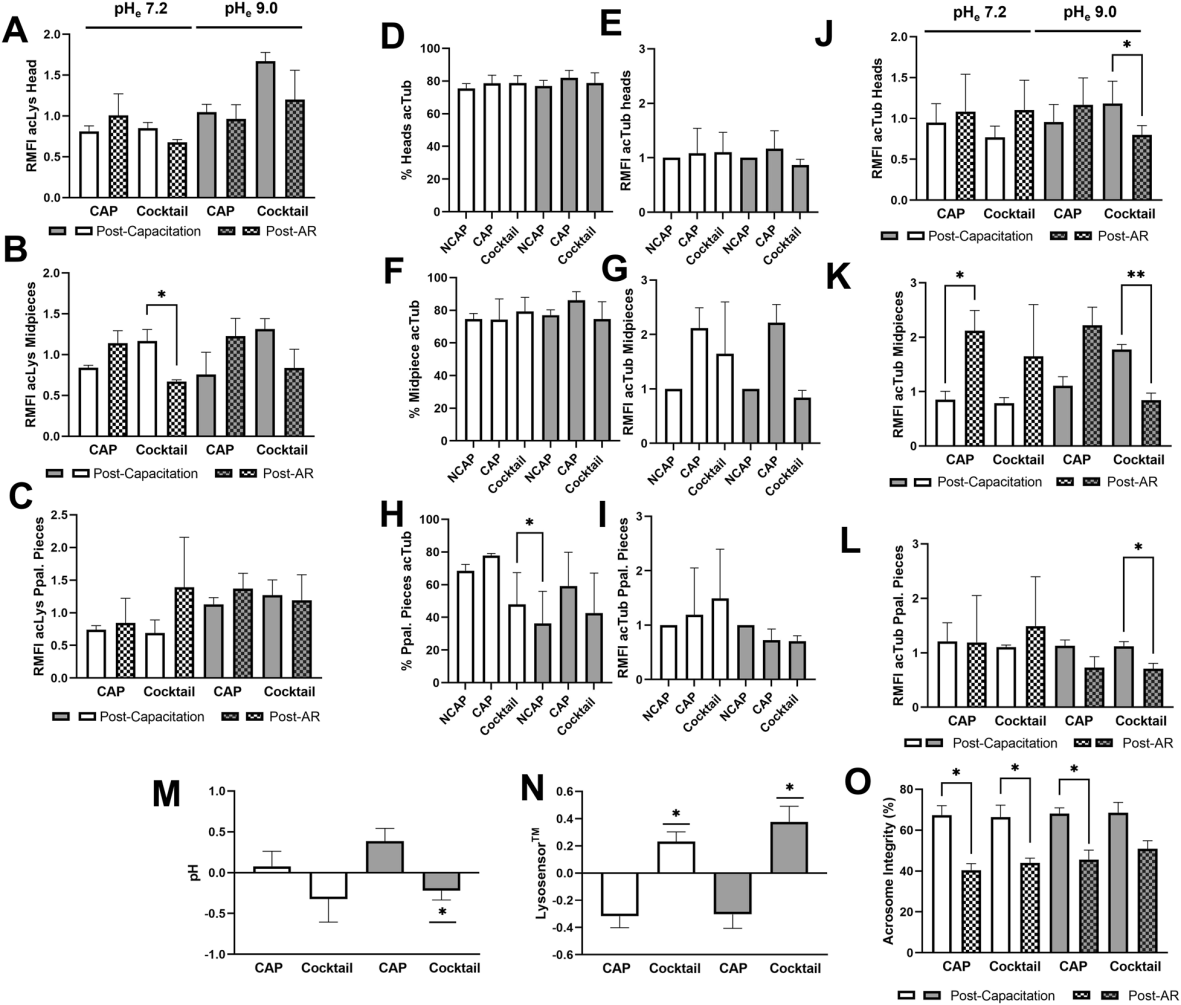
Differences of acetylated proteins (acLys), including acTub prior and post-AR of sperm incubated in capacitating (CAP) conditions, between the presence and the absence of the acetylase inhibitor cocktail (Cocktail) at pH 7.2 (white bars) and 9.0 (grey bars). For AR induction, after 3 h of incubation in the indicated conditions, sperm were exposed to progesterone (10 µM/mL) for further 10 min. Acetylation levels before and after the AR are shown for the **A** head, **B** midpiece, and **C** principal piece. Due to the observed reduction in acLys, acTub was assumed to be a candidate protein experiencing modifications post-AR. Then, acTub levels were evaluated and expressed as the percentage of sperm with acTub, and accumulation of acTub in the **D, E** head, **F, G** midpiece, and **H, I** principal piece. Differences of accumulated acTub before and after AR induction in the **J** head, **K** midpiece, and **L** principal piece. The accumulation of acTub is expressed as the fold change in mean fluorescence intensity (MFI) relative to the NCAP sample at each pH (assigned a value of 1; RMFI). Differences in **M** intracellular and **N** acrosomal pH after the AR and **O** reduction in acrosome integrity of sperm incubated under the specific conditions after inducing the AR. All columns show mean ± s.e.m. (* *P*<0.05)

Our focus shifted to the AR after identifying the interplay between acetylation dynamics, acetylase activity, and pH during capacitation. Briefly, following 3 h of incubation under the described experimental conditions, progesterone—which actively triggers the AR in vitro [32]—was added and sperm were further incubated for 10 min. Our results showed that, at pH 7.2, the exposure of capacitated sperm to progesterone reduced acrosome integrity compared to non-capacitated samples, irrespective of the activity of acyltransferases (*P* < 0.05). Under pH 9.0 and following exposure to progesterone, there were no differences in the acrosome integrity between treatments (Fig. 3A). We then evaluated acLys in different sperm regions after inducing the AR, and found that the percentage of viable sperm showing acLys in the head was significantly greater (*P* < 0.05) in sperm incubated in capacitating conditions at pH 7.2 than in those incubated in non-capacitating conditions at pH 7.2 and 9.0 (Fig. 3B). This comparison is shown in Fig. 4O, where we examined whether acrosome integrity was altered before and after progesterone exposure. In Fig. 3A, we compared acrosome integrity between different treatments following progesterone addition. The presence of acetylase inhibitors at pH 7.2 prevented the increase in acetylation, which is consistent with previous observations (Fig. 1H, K, N), further demonstrating the efficacy of the inhibitor cocktail under conditions that also induce the acrosome exocytosis. Additionally, regarding the accumulation of acetylated proteins after inducing the AR, the incubation in a capacitating medium at pH 9.0 resulted in a higher (*P* < 0.05) accumulation of acetylated proteins in the sperm midpiece compared to what was observed at pH 7.2 (Fig. 3F). No further differences were observed in the number of sperm developing acLys (Fig. 3C, D). Notably, the accumulation of acetylated proteins observed after capacitation, including those under conditions favoring non-enzymatic acetylation, was no longer detectable following AR induction. As a result, similar levels of acLys accumulation were observed across all sperm regions, with the exception of the midpiece of capacitated sperm at pH 7.2, where the accumulation was significantly higher than that seen under similar conditions at pH 9.0 (Fig. 3E–G).

The next step was assessing the cytoplasmic and acrosomal pH, given that alkalinization is a prerequisite for triggering the AR [13, 33]. After 3 h of incubation under capacitating conditions at pH 7.2, sperm showed significantly greater pH levels than non-capacitated sperm at the same pH (*P* < 0.05: Fig. 3H). The incubation of sperm at pH 9.0 with the cocktail of inhibitors increased the cytoplasmic pH compared to non-capacitated samples at pH 7.2 (*P* < 0.05; Fig. 3H). After the induction of the AR, these differences were no longer observed, and no significant differences were found between samples (Fig. 3I). Regarding acrosomal pH, following capacitation and the AR, incubation of sperm under capacitating conditions at pH 7.2 led to a significant increase in acrosomal pH (*P* < 0.05) compared to non-capacitated samples, as indicated by the reduced Lysosensor™ intensity, independent of acetylase activity (Fig. 3J, K). Notably, sperm incubated in the presence of the inhibitor cocktail exhibited even higher acrosomal pH than those incubated under non-capacitating conditions at pH 9.0. No other significant differences were observed between samples. Representative images of these cytoplasmic and acrosomal pH variations are shown in Fig. 3L–M.

### Acetyltransferase activity might participate in the pH maintenance of capacitated sperm for an effective AR

The assessment of acetylation dynamics during capacitation and the AR revealed distinct acetylation patterns between these two physiological events, suggesting a potential redistribution or deacetylation of acetylated proteins. Then, we aimed to compare the acetylation profiles of sperm that had achieved capacitation under the conditions proposed (i.e., after 3 h of incubation under the regimen described and before progesterone exposure) and those that had undergone the AR (following the incubation with progesterone). While the accumulation of acetylated proteins in the head and principal piece did not show significant variation from capacitation to the AR, the levels of acetylated proteins accumulated in the midpiece during capacitation in the presence of acetyltransferase inhibitors decreased significantly (*P* < 0.05) following the induction of the AR (Fig. 4A–C). As cytoskeletal proteins are known to undergo specific modifications before they can participate in triggering the AR [34, 35], we speculated that acTub could be a candidate among the acetylated proteins showing redistribution/deacetylation following AR induction. To investigate this, we first evaluated acTub levels after the addition of progesterone to samples. Our results showed that the percentage of viable sperm exhibiting acTub in the connecting piece, midpiece, and principal piece, and the accumulation of acTub in these regions did not significantly vary between samples (Fig. 4D–L). Nevertheless, the acTub accumulation in the midpiece during capacitation was higher under conditions supporting non-enzymatic acetylation than under most of the conditions under study (*P* < 0.05; Fig. 2G). We therefore examined whether this accumulated acTub degrades, redistributes, or instead persists or increases after inducing the AR with progesterone. Our results indicated that the acTub accumulated during capacitation under non-enzymatic conditions in the connecting piece, midpiece and principal piece of viable sperm decreased significantly (*P* < 0.05) after AR induction (Fig. 4J–L), suggesting that the intracellular or acrosomal pH post-AR failed to sustain non-enzymatic acetylation levels. Conversely, inducing the AR in those viable sperm capacitated under standard conditions significantly (*P* < 0.05) increased the acTub occurrence in their midpiece, indicating that constitutive α-tubulin is targeted for acetylation during the AR (Fig. 4K). We hypothesized that the reduction in acTub observed under conditions leading to non-enzymatic acetylation was due to the inability of sperm to maintain the intracellular and/or acrosomal pH levels achieved during capacitation until they underwent the AR. As shown in Fig. 4M, N, exposure of sperm to the acetyltransferase inhibitor cocktail significantly impaired their ability to maintain intracellular and acrosomal pH levels, regardless of the pH conditions. Consequently, the variation in intracellular and acrosomal pH between capacitated sperm and those that underwent the AR was significantly (*P* < 0.05) greater in sperm incubated with the cocktail than in those incubated without it. Given that the pHe was standardized to 7.2 and 9.0 before incubation, eliminating any potential acidification effect from the inhibitor cocktail, these results suggest the participation of enzymatic acetylation in the maintenance of pH during the AR. Particularly, at pH 9.0, the sperm ability to undergo the AR decreased, but the proportion of viable sperm with an intact acrosome was not significantly reduced unlike their counterparts (*P* < 0.05; Fig. 4O).

## Discussion

Protein acetylation at lysine residues is regulated by the interplay between KATs and KDACs [36], with non-enzymatic pathways facilitated by the alkaline pH also contributing to the cellular acetylation landscape [10]. While non-enzymatic acetylation in sperm was not explored before, the conditions encountered during capacitation, such as exposure to female reproductive fluids with increasing pH values, provide a conducive environment for these non-enzymatic events. Our findings now unravel that non-enzymatic acetylation can occur in sperm when exposed to alkaline pH conditions. We observed a significant increase in acLys in both the head and midpiece of sperm when KATs were inhibited and sperm were incubated at an alkaline pHe of 9.0. Although we cannot ascertain the complete inhibition of all KATs, the observed acLys accumulation would unlikely be due to non-inhibited KATs, as no similar enhancement was noted without inhibition (Fig. 1E, F; CAP at pH 9.0). This suggests that the pHi alkalinization observed—which was not achieved under capacitating conditions at pHe 9.0 (Fig. 3H)—is essential for non-enzymatic acetylation. These results provide the first evidence of non-enzymatic acetylation of proteins in sperm and, while representing an initial attempt, we propose a mechanistic model for pH-dependent acetylation of sperm proteins. Elevated pHe conditions encountered by sperm in the oviduct leads to pHi alkalinization, which enhances the activity of KDACs [37]. This results in an increased removal of acetyl groups from lysine residues on sperm proteins [8], which in turn generates more available lysine residues for subsequent acetylation. Concurrently, AcCo-A undergoes accelerated dissociation at alkaline pH [38] (Di Sabato et al., 1961), resulting in a higher concentration of free acetyl groups. The ε-amino groups of lysine residues, which are protonated at acidic and neutral pH [10], become deprotonated due to the alkalinization of pHi during the sperm journey through the oviduct, as observed in alkaline conditions [5], thereby increasing their nucleophilicity. This deprotonation enables lysine residues to act as nucleophiles and attack the electrophilic carbonyl group of AcCo-A [10]. Consequently, this nucleophilic attack enhances the acetylation of sperm lysine residues that have become available due to prior pH-dependent (i) deacetylation and (ii) increased concentration of free acetyl groups. Thus, the interplay between enhanced and alkaline-mediated (i) deacetylase activity, (ii) rapid AcCo-A dissociation, and (iii) lysine deprotonation might collectively promote the acetylation of sperm proteins with a minor contribution of KATs. It is also worth mentioning that AcCo-A is provided in sperm through the oxidation of pyruvate and lactate [39, 40] that, under our experimental conditions, were supplied by incubation media [41, 42], ensuring their consistent availability for the acetylation events observed.

The functions of non-enzymatic acetylation in cells remain largely unknown, despite the identification of several target proteins with known functions, including histones, albumin, and polylysine [43]. Here, we demonstrated that, in sperm, α-tubulin is one of the proteins undergoing acetylation under the conditions leading to non-enzymatic events. Specifically, we observed an accumulation of acTub in the midpiece of the sperm tail, the region where mitochondria are densely packed surrounding the microtubules of the tail axoneme. Notably, this modification in this particular region was correlated with an impaired switch to hyperactivated-like motility, while promoting slower movement. The involvement of non-enzymatic acetylation in regulating sperm motility is consistent with the biological significance of the establishment of the code of PTMs in a type of cells that are transcriptionally and translationally inactive [26]. This pH reactivity and its impact on motility may be crucial for sperm to rapidly adapt to pH fluctuations within the female reproductive tract towards the fertilization site. In our study, the association between acTub accumulation and diminished motility contrasted with previous findings linking it to enhanced microtubule stability and sperm motility [30, 44, 45], suggesting that non-enzymatic α-tubulin hyperacetylation is more related to mitochondrial function than to the structure of the axoneme. Supporting this notion, the lack of an enhanced acLys signal in the principal piece—where axonemal microtubules predominate in the absence of mitochondria—further reinforces the idea that acTub under conditions leading to non-enzymatic acetylation is more likely associated with the mitochondrial sheath than with the axonemal cytoskeleton. Consistent with this idea are data demonstrating that mitochondrial α-tubulin acetylation particularly affects energy production [46, 47], which is paramount for sperm motility [26]. Furthermore, it is clear that mitochondria, as the primary site of cellular Ac-CoA generation [26], provide an ideal environment for the non-enzymatic acetylation observed in the midpiece [48] and are the organelles where acetylation is more pronounced [49, 50]. These findings, therefore, suggest a specific role of mitochondrial acetylation in modulating sperm motility, highlighting the need for further investigation into how these processes may influence male fertility.

Beyond its established role in modulating sperm motility, we also investigated whether non-enzymatic acetylation exerts a functional influence after capacitation. Although this posttranslational modification proved sufficient to induce protein acetylation—most notably within the midpiece—during capacitation, it failed to sustain these acetylation marks over the AR. This loss is likely attributable to the sperm’s inability to maintain the elevated intracellular and acrosomal pH levels necessary to support non-enzymatic acetylation once capacitation has been achieved. In contrast, under standard capacitating conditions, acetylation was maintained or even enhanced following AR induction, implicating enzymatic mechanisms—rather than purely pH-driven chemical processes—in the stabilization of acetylation throughout the AR. On the contrary, our results uncovered that acetylase activity may play a role in maintaining the pHi during capacitation and until the AR occurs. Specifically, the inhibition of acetylase activity was observed to compromise the maintenance of the intracellular and acrosomal pHs levels achieved during capacitation, as evidenced by a significant reduction in intracellular and acrosomal pH after the AR, even at pHe 9.0. This reduction in the two pHs was associated with (i) a diminished ability to trigger the AR, and (ii) a notable loss of acetylated proteins, including acTub. The latter could indicate that acetylation events no longer occurred, resulting in a shift in the balance between acetylation and deacetylation towards increased deacetylation. Consequently, this protein deacetylation could contribute to the continuous production of acetyl groups in the form of acetic acid, which dissociates into acetate and hydrogen ions thereby contributing to the acidification of the pH [51]. In somatic cell lines, such as HeLa, MDA-MB-468, and other cancer cells, acetylation acts as a regulator of pHi, serving as a crucial mechanism for their survival [37]. This fact suggests that conserved mechanisms have evolved across diverse cellular types to manage the pH fluctuations throughout their lifespan, ensuring effective functionality and overall cellular health.

In conclusion, using a proof-of-concept approach, we identified and characterized non-enzymatic protein acetylation in sperm—a previously unreported molecular event. Our findings demonstrated that alkaline pHi can drive selective acetylation of α-tubulin, regulating motility while having a more limited effect on the AR. We also highlight the importance of intracellular and acrosomal pH, as well as head protein acetylation, in facilitating the AR, and suggest a potential role for acetyltransferases in pH regulation for an effective AR. Although preliminary, our data indicate that an imbalance between enzymatic and non-enzymatic acetylation may restrict the proportion of sperm reaching full fertilizing competence. It is plausible that only those sperm maintaining an optimal acetylation profile—regulated by both mechanisms and marked by tubulin acetylation in the midpiece—are able to fertilize the oocyte. Future investigations should aim to elucidate further this mechanism under conditions that, as pH values within the near-physiological range (e.g., 7.8–8.0), more closely mimic the physiological milieu, as this may help determine the biological relevance of our findings. 

## Supplementary Information

Additional file 1

Additional file 2

## Data Availability

Data supporting the findings of this study are available from the corresponding authors upon reasonable request.
