# Supplementary material for: Decoding a novel non-enzymatic protein acetylation mechanism in sperm that is essential for fertilizing potential
Source: Biol Res. 2025 May 29;58:30. doi: 10.1186/s40659-025-00613-6 (PMC12121157; doi:10.1186/s40659-025-00613-6)
Supplement: Supplementary file 1 — Additional file 1 [file 40659_2025_613_MOESM1_ESM.docx]

**Table 1**. Intracellular pH values of sperm at the beginning of the incubation period under the studied conditions.

| **pHe 7.2** | | | **pHe 9** | | |
| --- | --- | --- | --- | --- | --- |
| **NC** | **CAP** | **Cocktail** | **NC** | **CAP** | **Cocktail** |
| 7.18±0.06 | 7.21±0.1 | 7.23±0.09 | 7.17±0.05 | 7.21±0.07 | 7.19±0.04 |


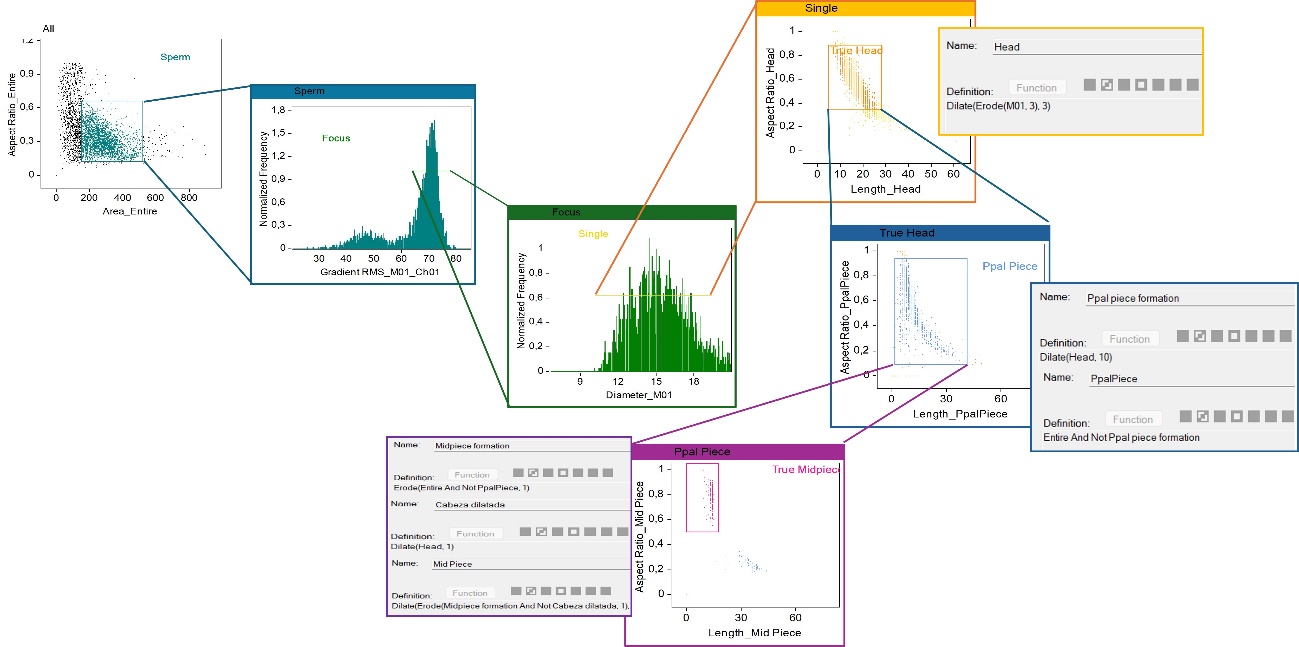


**Figure 1**. The gating approach for evaluating IF samples using image-based flow cytometry (AMNIS) involved using aspect ratio and area to distinguish sperm populations. Standard focus and single-cell gating calculations were then performed with IDEAS software. Subsequently, several masks were created to specifically analyze the sperm head, midpiece, and principal piece of the flagellum, as detailed in the figure.


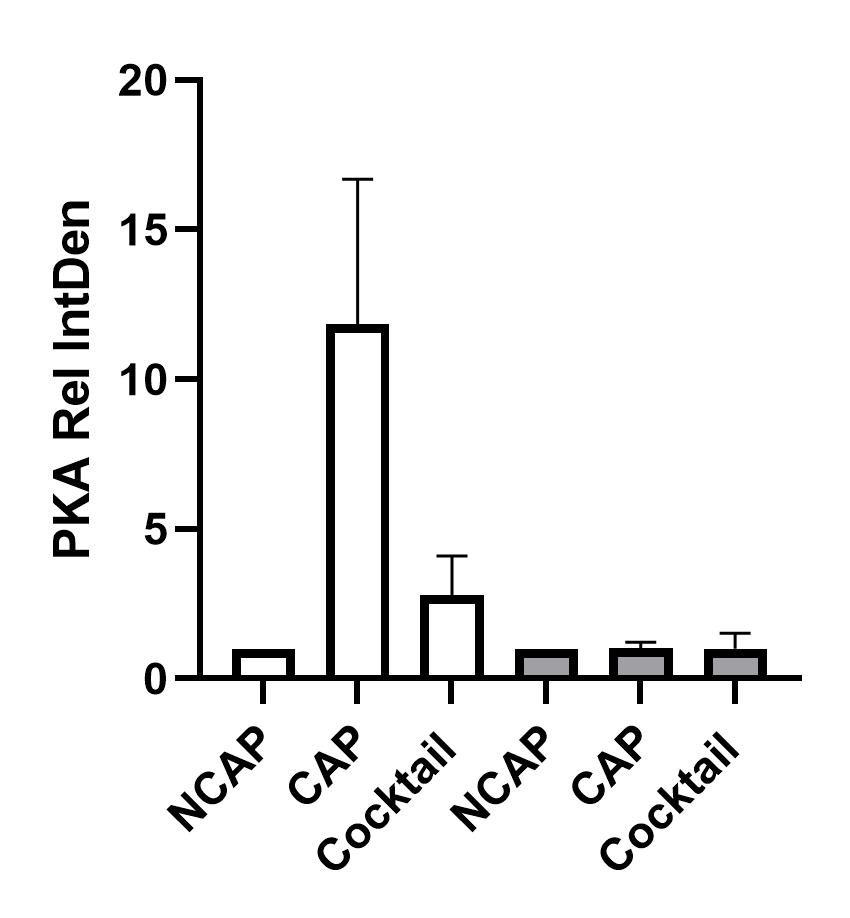

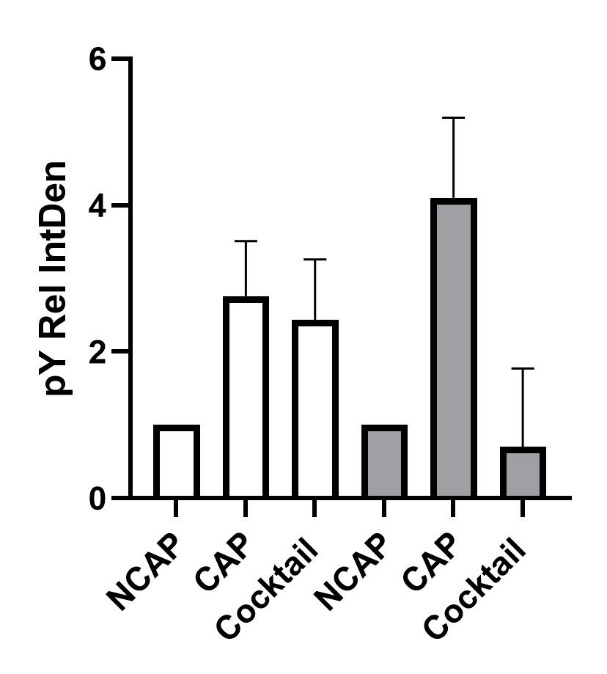


**Figure 2**. Densitometric results of Western blotting with anti-protein kinase A (PKA) and anti-phosphotyrosine (pY) following incubation of sperm under non-capacitating (NCAP) and capacitating (CAP) conditions, as well as under CAP conditions with an acetylase inhibitor cocktail (Cocktail), at pH 7.2 (white bars) and pH 9 (grey bars). All columns show mean ± s.e.m.
