# Supplementary figures and images for: Decoding a novel non-enzymatic protein acetylation mechanism in sperm that is essential for fertilizing potential
Source: Biol Res. 2025 May 29;58:30. doi: 10.1186/s40659-025-00613-6 (PMC12121157; doi:10.1186/s40659-025-00613-6)

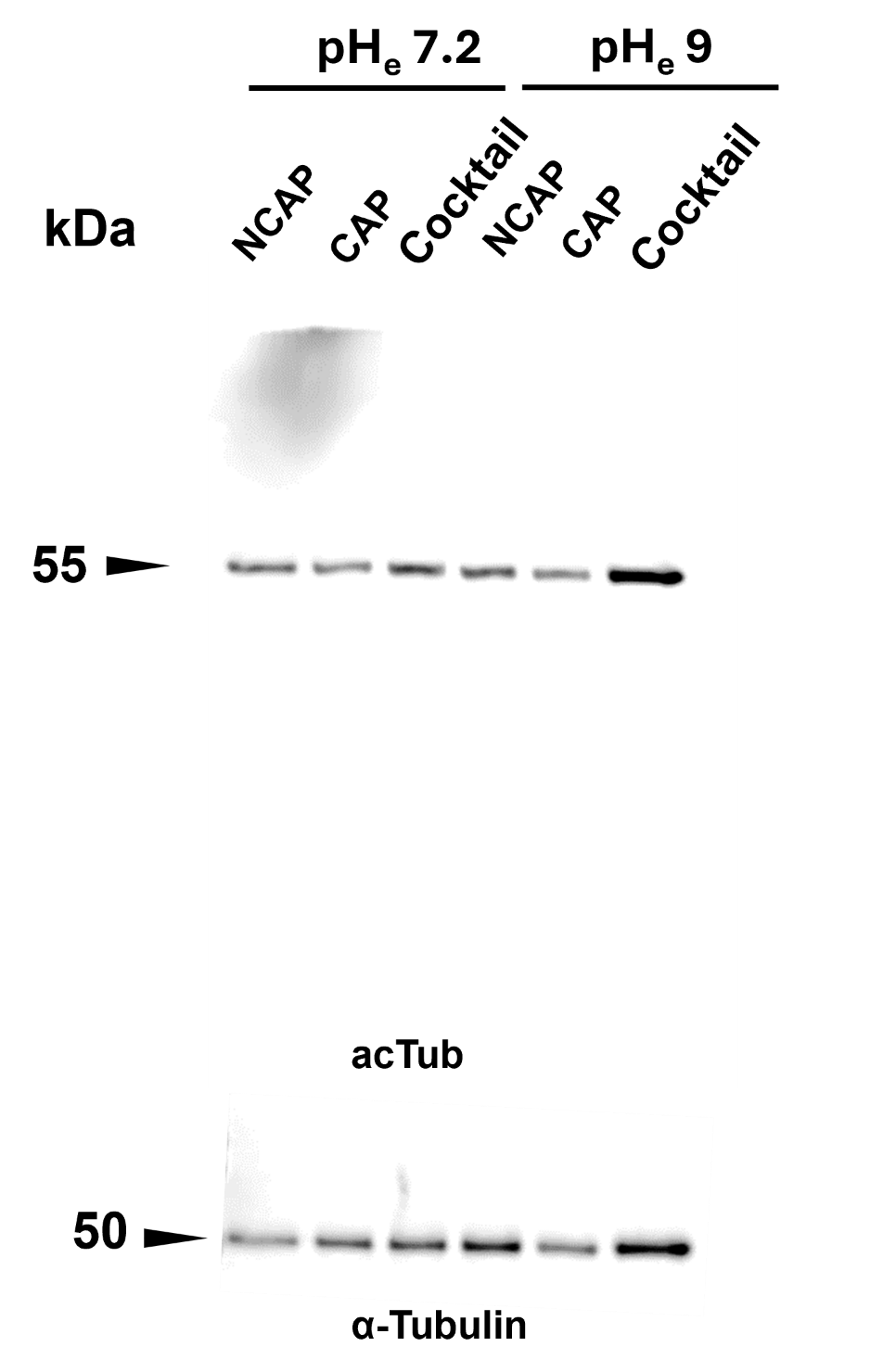


**Figure 1**. Full uncropped Blot images of acetylated tubulin (acTub).

Supplement: Supplementary file 2 — Additional file 2 [file 40659_2025_613_MOESM2_ESM.docx]
